# Diagnostic accuracy of the Enferplex Bovine Tuberculosis antibody test in cattle sera

**DOI:** 10.1038/s41598-023-28410-9

**Published:** 2023-02-01

**Authors:** Amanda O’Brien, John Clarke, Alastair Hayton, Andy Adler, Keith Cutler, Darren J. Shaw, Clare Whelan, Neil J. Watt, Gordon D. Harkiss

**Affiliations:** 1 Enfer Scientific, Unit T, M7 Business Park, Newhall, Naas, County Kildare Ireland; 2 SureFarm Ltd, The Transmission Hall, Rampisham Business Centre, Rampisham Down, Maiden Newton, Dorset DT2 0HS UK; 3 Royal (Dick) School of Veterinary Studies & The Roslin Institute, University of Edinburgh, Easter Bush Campus, Edinburgh, UK; 4 MV Diagnostics Ltd, Roslin Innovation Centre, University of Edinburgh, Easter Bush Campus, Edinburgh, UK

**Keywords:** Infection, Immunology, Diseases

## Abstract

Bovine tuberculosis is a contagious bacterial disease of worldwide economic, zoonotic and welfare importance caused mainly by *Mycobacterium bovis* infection. Current regulatory diagnostic methods lack sensitivity and require improvement. We have developed a multiplex serological test for bovine tuberculosis and here we provide an estimate of the diagnostic accuracy of the test in cattle. Positive and negative reference serum samples were obtained from animals from Europe and the United States of America. The diagnostic specificity estimate was 98.4% and 99.7% using high sensitivity and high specificity settings of the test respectively. Tuberculin boosting did not affect the overall specificity estimate. The diagnostic sensitivity in samples from *Mycobacterium bovis* culture positive animals following tuberculin boosting was 93.9%.The relative sensitivity following boosting in tuberculin test positive, lesion positive animals and interferon gamma test positive, lesion positive animals was 97.2% and 96.9% respectively. In tuberculin test negative, lesion positive animals and in interferon gamma test negative, lesion positive animals, the relative sensitivity following tuberculin boosting was 88.2% and 83.6% respectively. The results show that the test has high diagnostic sensitivity and specificity and can detect infected animals that are missed by tuberculin and interferon gamma testing.

## Introduction

Despite official control programmes, bovine tuberculosis (bTB) continues to be a serious economic, zoonotic and welfare problem worldwide. Failure to detect infected animals due to the poor sensitivity of officially approved diagnostic tests is thought to be an important contributing factor^1–5^. No gold standard diagnostic tests exist for bTB. Culture of *Mycobacterium bovis (M. bovis)* from post-mortem tissue is definitive proof of infection, though the sensitivity of bacterial isolation from tissues varies from 58.0 to 80.0%^6,7^. In some countries, the presence of visible lesions (VL) typical of bTB at post-mortem is taken as evidence of *M. bovis* infection.

Tuberculin tests (TT) are approved for disclosure of infected herds and animals by the World Organisation for Animal Health (WOAH), formerly Office International des Epizooties (OIE) and the European Union (EU). Given the high specificity (99.98%) reported for the Single Intradermal Comparative Cervical Tuberculin test (SICCT)^8^, a positive result in this test is regarded as very good indirect evidence of infection, though the reported sensitivity varies from 40.0 to 100%^4,5,9–12^. The Single Intradermal Cervical tuberculin test (SICT) and the Caudal Fold Tuberculin test (CFT) are approved by the WOAH and used in many countries as primary screening tests. The specificity estimates for the CFT range from 89.2 to 99.0% with a sensitivity ranging between 63.2 and 93.0%^9,13^. The SICT specificity varies between 53.1 and 99.0%^9,14,15^. The sensitivity estimates of the SICT range from 80.2 to 100%^9^, though Bayesian analysis yielded a range from 53.0 to 69.4%^15^.

The interferon gamma (IFNγ) test is WOAH validated and accepted as a supplemental test by the EU. The test has higher sensitivity estimates compared to the SICCT, ranging from 67.0 to 85.8%^4,5,9^, but has a lower specificity (96.6%: range 85.0–99.6%) than the SICCT^9,15,16^.

Recently, serology tests have been evaluated for their ability to detect infected animals^17–23^. These studies have shown that antibody responses can appear as early as 3–8 weeks post infection and challenge the view that such responses only appear in the late phases of the disease and have low sensitivity^9^. Several studies have shown that antibody responses are boosted following injection of tuberculin, with the optimum time for anamnestic responses to be detected occurring within a few days and lasting up to 30 days or longer post TT^19,21–27^. The above studies suggest that serology tests could play a useful role in detecting and controlling bTB.

We have developed a sensitive chemiluminescent multiplex serology test—the Enferplex Bovine TB antibody test (Enferplex bTB test)—for detecting antibodies in bTB infected cattle based on an array of antigens printed onto the surface of microtitre plates. Responses to individual antigens in the Enferplex bTB test can be detected and quantified. The results of using prototype versions of the methodology have been published previously^10,28–31^. Here we describe an assessment of the diagnostic performance the Enferplex bTB test using 11 target antigens with a view to assessing its possible value in aiding the control of bTB in cattle. The specific aims were to (a) determine the test sensitivity and specificity using reference samples from the EU and the USA, with and without anamnestic boosting of antibody responses by injection of partially purified derivative of bovine tuberculin (PPDb), and (b) investigate the ability of the test to detect infected animals missed by cell-mediated diagnostic tests.

## Results

### Estimates of diagnostic specificity and sensitivity of the Enferplex Bovine TB antibody test

Negative and positive references samples used in the study to estimate diagnostic specificity and sensitivity are shown in Tables 1 and 2 respectively.

**Table 1:** Summary of negative reference serum samples.

^a^Origin of samples	Number of samples	Number of herds	Age (Years) M +/− SD	^d^Reference standard
UK Set 1	1150	72	4.9 ± 2.1	England, SICCT tested every 4 years: SICCT negative > 8 years; no bTB breakdowns within 10 km in previous 12 months. 1130 MAP ELISA negative; 20 MAP ELISA positive. Non-boosted
UK Set 2	1129	14	5.6 ± 3.16	Scotland OTF status, slaughterhouse surveillance,1118 MAP ELISA negative, 11 MAP positive. Non-boosted
UK Set 3	183	3	^b^NK	England, SICCT tested every 4 years; SICCT negative > 8 years, no bTB breakdowns within 10 km in previous 12 months. Boosted
IE Set 1	204	37	3.8 ± 3.0	SICCT negative, IFNγ negative herds from low bTB prevalence areas MAP antibody negative; 1 MAP culture positive
IE Set 2	439	66	^b^NK	SICCT negative, from low bTB prevalence areas. 300 MAP antibody negative; 139 MAP antibody positive
CH/LI	554	33	^c^2.4 ± 0.6(n = 133)	OTF country status, SICCT negative, no recent history of bTB
NO Set 1	248	13	^c^3.9 ± 1.6(n = 210)	OTF country status, slaughterhouse surveillance, no bTB for > 20 years, MAP antibody negative
NO Set 2	190	8	^b^NK	OTF status, slaughterhouse surveillance, no bTB for > 20 years, 182 MAP antibody negative; 3 MAP antibody positive; 5 unknown MAP status
USA Set 1	336	^b^NK	3.3 ± 2.0	CFT test negative from bTB free herds from 11 States. 335 MAP antibody negative; 1 MAP antibody positive.
USA Set 2	45	^b^NK	^b^NK	CFT negative from bTB free herds, 45 MAP culture positive; 43 MAP antibody positive
NL	100	^b^NK	^b^NK	OTF country status, slaughterhouse surveillance, 100 MAP culture positive; 33 MAP antibody positive
ES	10	^b^NK	^b^NK	OTF herd status, no recent history of bTB, slaughterhouse surveillance, MAP lesion positive at post-mortem, 10 MAP antibody positive
FR Set 1	3	^b^NK	^b^NK	OTF country status, SICT negative, no recent history of bTB, 3 MAP culture positive
FR Set 2	161	1	^b^NK	OTF country status, SICT negative, no recent history of bTB. Boosted
Total	4752	247	5.2 ± 2.8	Bovine TB free

^a^Serum samples were obtained from bTB free herds in the United Kingdom (UK), Ireland (IE), Switzerland/Liechtenstein (CH/LI), Norway (NO), United States of America (USA), Netherlands (NL), Spain (ES) and France (FR). ^b^Not known; ^c^Age data not complete (number of animals); ^d^SICCT test (UK, IE, CH/LE); CFT test (USA); SICT test (ES, FR); OTF—Officially Tuberculosis Free; MAP—*Mycobacterium avium* subsp. *paratuberculosis.*

**Table 2:** Summary of positive field reference serum samples.

^a^Origin of samples	Number of herds	Age in years Mean +/− SD	Number of samples				
			Comparator				
			^c^TT positive	^c^TT inconclusive reactor	IFNγ positive	Lesion positive	*M. bovis* culture positive
UK	746	3.9 +/− 2.6	^d^676	^d^676	1307	453	218
IE	550	4.2 +/− 2.7	^d^1,411	6	1302	933	20
IT	^b^NK	^b^NK	^e^15	–	110	54	88
USA	^b^NK	4.2 +/− 3.6 (n = 113)	^f^91	–	34	156	156
Total	1296	4.1 + /− 3.0	2193	682	2753	1596	482

^a^Serum samples were obtained from bTB breakdown herds in the United Kingdom (UK), Ireland (IE), Italy (IT), and United States of America (USA). The comparators used for validation were tuberculin tests, tuberculin test inconclusive reactors, IFNg test, lesions at post-mortem, and culture of *M. bovis* from post-mortem tissues. The total number of samples tested using the Enferplex bTB test for each comparator is shown in the last row of the Table.

^b^NK—not known. ^c^TT—tuberculin test. ^d^SICCT; ^e^SICT; ^f^CFT.

#### Diagnostic specificity

In non-boosted animals, the overall diagnostic specificity was 98.4% (95% CI:98.0–98.7%) and 99.7% (95% CI: 99.5–99.8%) at the high sensitivity (Hse) and high specificity (Hsp) settings of the test respectively (Table 3 and Supplementary Table S1 online). In boosted samples from bTB free herds, the diagnostic specificity was 98.8% (95% CI: 97.0–99.5%) and 99.7% (95% CI: 97.4–99.8%) at the Hse setting and Hsp setting of the test respectively.

**Table 3:** Diagnostic specificity estimates of the Enferplex Bovine TB antibody test.

Animal category under evaluation	Sample boost status and source	Statistical variable	High sensitivity setting	High specificity setting
bTB free	Non boosted UK, IE, NO, NL, CH/LE, ES, USA	N DSp CI	4408 98.4% 98.0–98.7	4408 99.7% 99.5–99.8
bTB free	Boosted UK, FR	N DSp CI	344 98.8% 97.1–99.6	344 99.1% 97.4–99.7
bTB free, MAP culture positive	Non-boosted NL, IE, ES, USA	N DSp CI	257 98.4% 96.1–99.4	257 100% –
bTB free, MAP ELISA positive	Non-boosted UK, IE, NO, NL, USA	N DSp CI	251 98.4% 96.0–99.4	251 100% –

Diagnostic specificity was estimated at the high sensitivity and high specificity settings using reference sera from bTB free animals with and without tuberculin boosting and with and without MAP infection. Boosted- samples taken within anamnestic window; non-boosted—samples taken out with the anamnestic window (5–30 days post tuberculin injection). N = number of samples; DSp—diagnostic specificity; CI—95% Confidence Interval.

To assess the diagnostic specificity in bTB-free cattle infected with *Mycobacterium avium* subsp*. paratuberculosis* (MAP), samples were tested from MAP culture positive animals and MAP ELISA positive animals. The results showed that at the Hse setting, the specificity was 98.4% in both MAP culture positive and MAP ELISA positive animals respectively. The equivalent estimates obtained at the Hsp setting were both 100%. These specificity estimates are close to the specificity estimates observed in bTB free cattle, indicating that MAP infection had no significant effect on the specificity of the Enferplex bTB test. The differences in diagnostic specificity observed in samples from different countries were small (Supplementary Table S2 online).

#### Diagnostic sensitivity

The diagnostic sensitivity estimates obtained for the Enferplex bTB test are shown in Table 4 and Supplementary Table S1 online.

**Table 4:** Diagnostic sensitivity estimates of the Enferplex Bovine TB antibody test.

Animal category under evaluation	Samples and source^a^	Statistical variable	High sensitivity setting	High specificity setting
*M. bovis* culture positive	Boosted samples UK, IE	N DSe CI	214 93.9% 89.9–96.4	214 93.9% 89.9–96.4
*M. bovis* culture positive	Non-boosted samples UK, USA	N DSe CI	179 76.0% 69.2–81.7	179 71.5% 64.5–77.6
*M. bovis* culture positive	Unknown boost status IT, IE	N DSe CI	89 80.9% 71.5–87.7	89 75.3% 65.4–83.1
*M. bovis* culture positive	All samples UK, IE, IT, USA	N DSe CI	482 84.9% 81.4–87.8	482 82.2% 78.5–85.3
SICCT positive	Boosted UK, IE	N RSe CI	1979 94.3% 93.1–95.2	1979 91.9% 90.6–93.0
SICCT positive or CFT positive	Non-boosted UK, IE, USA	N RSe CI	229 77.7% 71.9 – 82.6	229 72.5% 66.4 – 77.9
SICT positive	Unknown boost status IT	N RSe CI	15 73.3% 48.1 – 89.1	15 66.7% 41.7 – 84.8
SICCT, SCIT or CFT positive	All samples UK, IE, IT, USA	N RSe CI	2193 92.4% 91.2–93.4	2193 89.7% 88.4–90.9
2 × IR positive	Boosted UK	N RSe CI	401 81.1% 76.9 – 84.6	401 75.1% 70.6–79.0
2 × IR positive	Non boosted UK	N RSe CI	57 56.1% 43.3 – 68.2	57 52.6% 39.9 – 65.0
2 × IR positive	All samples UK	N RSe CI	458 78.0% 73.9–81.5	458 72.3% 68.0–76.2
1 × IR positive	Boosted UK	N RSe CI	134 85.8% 78.9–90.7	134 80.6% 73.1–86.4
1 × IR positive	Non boosted UK	N RSe CI	85 43.5% 33.5–54.1	85 40.0% 30.2–50.6
1 × IR positive	All samples UK	N RSe CI	219 69.4% 63.0–75.1	219 64.8% 58.3–70.9
IFNγ test positive	Boosted samples UK, IE	N RSe CI	1341 90.0% 88.3–91.5	1341 85.6% 83.6–87.4
IFNγ test positive	Non-boosted samples UK, USA	N RSe CI	1297 30.5% 28.0–33.0	1297 25.0% 22.7–27.4
IFNγ test positive	Unknown boost status IT, IE	N RSe CI	115 67.0% 57.9–74.9	115 64.4% 55.3–72.5
IFNγ test positive	All samples UK, IE, IT, USA	N RSe CI	2753 61.0% 59.2–62.8	2753 56.2% 54.3–58.0

Diagnostic sensitivity was estimated at the high sensitivity and high specificity settings using reference sera from animals positive by *M. bovis* culture, and relative sensitivity was similarly estimated from animals positive by SICCT, CFT, IFNγ test or SICCT inconclusive reactor (IR). ^a^Boosted—samples taken within anamnestic window (5–30 days post tuberculin injection); non-boosted—samples taken outwith the anamnestic window; United Kingdom (UK), Ireland (IE), Italy (IT); United States of America (USA). ^b^N = number of samples; DSp—diagnostic specificity; DSe—diagnostic sensitivity; RSe—relative sensitivity; CI—95% Confidence Interval.

#### *M. bovis* culture comparator

The results show that in animals that received a tuberculin boost, the diagnostic sensitivity in *M. bovis* culture positive animals was 93.9% (95% CI: 89.9–96.4%) and 93.9% (95% CI: 89.9–96.4%) using the Hse and Hsp settings respectively, while in non-boosted animals the sensitivity was 76.0% (95% CI: 69.2–81.7%) and 71.5% (95% CI: 64.5–77.6%) at the Hse and Hsp settings respectively. The differences between boosted and non-boosted samples were statistically significant (*P* < 0.001 in both cases). Kappa analysis using boosted samples and negative controls gave kappa values of 0.820 (95% CI: 0.782–0.857) and 0.934 (95% CI: 0.909–0.959) at the Hse and Hsp setting respectively, indicating almost perfect agreement in both cases.

#### SICCT comparator

The Rse of the test using samples from SICCT positive animals was significantly higher statistically at 94.3% (95% CI: 93.1–95.2%) and 91.9% (95% CI: 90.6- 93.0%) using boosted samples compared with 77.7% (95% CI: 71.9–82.6%) and 72.5% (95% CI: 66.4–77.9%) in SICCT positive non-boosted samples using the Hse and Hsp settings respectively (*P* < 0.001 in both cases). Kappa analysis using boosted samples and negative controls gave kappa values of 0.932 (95% CI: 0.923–0.942) and 0.935 (95% CI: 0.925–0.945) at the Hse and Hsp setting respectively, indicating almost perfect agreement in both cases.

In animals that received a tuberculin boost and showed two consecutive SICCT inconclusive reactions (2 × IR), the Rse was 81.1% (95% CI: 76.9–84.6%) and 75.1% (95% CI: 70.6–79.0%) at the Hse and Hsp settings respectively. In non-boosted samples, the Rse was significantly lower at 56.1% (95% CI: 43.3–68.2%) and 52.6% (95% CI: 39.9–65.0%) using the Hse and Hsp setting respectively (*P* < 0.001 in both cases). Kappa analysis using boosted samples and negative controls gave kappa values of 0.800 (95% CI: 0.769–0.832) and 0.828 (95% CI: 0.797–0.859) at the Hse and Hsp setting, indicating substantial and almost perfect agreement respectively. There were 16 animals that were VL or *M. bovis* culture positive in the 2 × IR sample set—13 boosted samples and 3 non-boosted samples. The Enferplex bTB test was positive in 13/13 boosted samples (100%) and in 2/3 non-boosted samples (66.7%) at both the Hse and Hsp settings.

In animals that received a tuberculin boost and showed an isolated SICCT IR (1 × IR), 85.8% (95% CI: 78.9–90.7%) and 80.6% (95% CI: 73.1–86.4%) were positive at the Hse and Hsp settings respectively. In non-boosted samples, 43.5% (95% CI: 33.5–54.1%) and 40.0% (95% CI: 30.2–50.6%) were positive using the Hse and Hsp setting respectively (*P* < 0.001 in both cases). Kappa analysis using boosted samples and negative controls gave kappa values of 0.716 (95% CI: 0.659–0.772) and 0.839 (95% CI: 0.790–0.888) at the Hse and Hse setting, indicating substantial and almost perfect agreement respectively. There were 14 animals that were VL or *M. bovis* culture positive in the 1 × IR sample set—3 boosted samples and 11 non-boosted samples. The Enferplex bTB test was positive in 3/3 boosted samples (100%) and in 5/11 non-boosted samples (45.5%) at both the Hse and Hsp settings.

#### IFNγ comparator

In samples from IFNγ positive animals that received a tuberculin boost, the Rse was significantly higher at 90.0% (95% CI: 88.3–91.5%) and 85.6% (95% CI: 83.6–87.4%) compared with 30.5% (95% CI: 28.0–33.0%) and 25.0% (95% CI: 22.7–27.4%) in non-boosted animals using the Hse and Hsp settings of the test respectively (*P* < 0.001 in both cases). Kappa analysis using boosted samples and negative controls gave kappa values of 0.899 (95% CI: 0.886–0.913) and 0.894 (95% CI: 0.880–0.908) at the Hse and Hse setting respectively, indicating almost perfect agreement in both cases.

### Estimates of relative sensitivity of the Enferplex Bovine TB antibody test in TT positive animals with and without lesions

The Rse of the test was assessed in samples from SICCT or CFT positive animals with and without bTB lesions. Serum samples from SICCT positive animals from UK and IE, and CFT positive animals from the USA, were tested using the Hse and Hsp settings of the test. The results obtained overall and in subsets relating to whether the samples were taken during the anamnestic window or not are shown in Table 5 and Supplementary Table S1 online.

**Table 5:** Relative sensitivity of the Enferplex Bovine TB antibody test in TT positive animals with and without lesions.

Animal category under evaluation		Samples and source^a^	Statistical variable^b^	High sensitivity setting	High specificity setting
SICCT positive	bTB lesion positive	Boosted UK, IE	N RSe CI	1024 97.2% 96.0–98.0	1024 96.5% 95.2–97.5
SICCT positive or CFT positive	bTB lesion positive	Non-boosted UK, IE, USA	N RSe CI	147 86.4% 79.9–91.0	147 81.0% 73.9–86.5
SCIT positive	bTB lesion positive	Unknown boost status IT	N RSe CI	6 100% –	6 83.3% 43.7–97.0
TT positive	bTB lesion positive	All samples UK, IE, IT, USA	N RSe CI	1177 95.8% 94.5–96.8	1177 94.5% 93.0–95.7
SICCT positive	bTB lesion negative	Boosted UK, IE	N RSe CI	909 91.9% 89.1–92.8	909 86.8% 84.4–88.9
SICCT positive	bTB lesion negative	Non-boosted UK, IE	N RSe CI	68 72.1% 60.4–81.3	68 66.2% 54.3–76.3
SICT positive	bTB lesion negative	Unknown boost status IT	N RSe CI	7 57.1% 25.0–84.2	7 57.1% 25.0–84.2
TT positive	bTB lesion negative	All samples UK, IE, IT	N RSe CI	984 89.5% 87.5–91.3	984 85.2% 82.8–87.2
SICCT negative	bTB lesion positive	Boosted UK, IE	N RSe CI	127 88.2% 81.4–92.7	127 81.9% 74.3–87.6
SICCT negative	bTB lesion positive	Non-boosted UK, IE, USA	N RSe CI	128 50.8% 42.2–59.3	128 44.5% 36.2–53.2
SICT negative	bTB lesion positive	Unknown boost status IT	N RSe CI	11 36.4% 15.2–64.6	11 36.4% 15.2–64.6
TT negative	bTB lesion positive	All samples UK, IE, IT, USA	N RSe CI	266 68.1% 62.2–73.4	266 62.0% 56.1–67.7

Diagnostic sensitivity was estimated at the high sensitivity and high specificity settings using reference sera from SICCT positive animals showing bTB lesions at post-mortem. ^a^Boosted- samples taken within anamnestic window (5–30 days post tuberculin injection); non-boosted—samples taken outwith the anamnestic window; United Kingdom (UK), Ireland (IE), Italy (IT); United States of America (USA). ^b^N = number; RSe—relative sensitivity; CI—95% Confidence Interval.

#### SICCT positive, lesion positive animals

The Rse observed in samples from SICCT positive animals with VL which were pre-boosted with PPDb was 97.2% (95% CI: 96.0–98.0%) and 96.5% (95% CI: 95.2–97.5%) at the Hse and Hsp settings respectively. In non-boosted samples, the equivalent figures were statistically significantly lower at 86.4% (95% CI: 79.9–91.0%) and 81.0% (95% CI: 73.9–86.5% respectively (*P* < 0.001). Kappa analysis using boosted samples and negative controls gave kappa values of 0.941 (95% CI: 0.930–0.953) and 0.970 (95% CI: 0.961–0.978) at the Hse and Hsp setting respectively, indicating almost perfect agreement in both cases.

#### SICCT positive, lesion negative animals

The results also show that the relative sensitivity of the test in SICCT positive boosted animals that show no visible lesions at post-mortem was 91.1% (95% CI: 89.1–92.8%) and 86.8% (95% CI: 84.4–88.9%) at the Hse and Hsp settings of the test respectively. Kappa analysis using boosted samples and negative controls gave kappa values of 0.899 (95% CI: 0.884–0.915) and 0.907 (95% CI: 0.891–0.922 at the Hse and Hsp setting respectively, indicating almost perfect agreement in both cases.

#### SICCT negative, lesion positive animals

Samples from animals from SICCT negative animals with lesions that were pre-boosted with PPDb had a Rse of 88.2% (95% CI: 81.4–92.7%) and 81.9% (95% CI: 74.3–87.6%) at the Hse and Hsp settings respectively. In non-boosted samples, the figures were statistically significantly lower at 50.8% (95% CI: 42.2–59.3) and 44.5% (95% CI: 36.2–53.2) respectively (*P* < 0.001 in both cases). Kappa analysis using boosted samples and negative controls gave kappa values of 0.716 (95% CI: 0.658–0.773) and 0.845 (95% CI: 0.796–0.894) at the Hse and Hsp setting, indicating substantial and almost perfect agreement respectively.

### Estimates of relative sensitivity of the Enferplex Bovine TB antibody test in IFNγ test positive animals with and without visible lesions

The Rse of the test was assessed in samples from animals with and without bTB lesions that were tested using the IFNγ test. Serum samples from IFNγ tested animals from UK, IE, IT and USA were assessed using the Hse and Hsp settings of the antibody test. The results obtained overall and in subsets relating to whether the samples were taken during the anamnestic window or not are shown in Table 6 and Supplementary Table S1 online.

**Table 6:** Relative sensitivity of Enferplex Bovine TB antibody test in IFNγ positive animals with or without visible lesions.

Animal category under evaluation		Samples and source^a^	Statistical variable^b^	High sensitivity setting	High specificity setting
IFNγ positive	bTB VL positive	Boosted UK, IE	N RSe CI	352 96.9% 94.5–98.3	352 95.5% 92.7–97.2
IFNγ positive	bTB VL positive	Non-boosted UK, IE, USA	N RSe CI	166 57.8% 50.2–65.1	166 51.8% 44.3–59.3
IFNγ positive	bTB VL positive	Unknown boost status IT, USA	N RSe CI	27 74.1% 55.3–86.8	27 74.1% 55.3–86.8
IFNγ positive	bTB VL positive	All samples UK, IE, IT, USA	N RSe CI	545 83.9% 80.5–86.7	545 81.1% 77.6–84.2
IFNγ positive	bTB VL negative	Boosted UK, IE	N RSe CI	936 88.5% 86.3–90.4	936 82.9% 80.4–85.2
IFNγ positive	bTB VL negative	Non-boosted UK, IE	N RSe CI	792 32.8% 29.7–36.2	792 26.6% 23.7–29.8
IFNγ positive	bTB VL negative	Unknown boost status IT	N RSe CI	16 18.8% 6.6–43.0	16 18.8% 6.6–43.0
IFNγ positive	bTB VL negative	All samples UK, IE, IT, USA	N RSe CI	1744 62.6% 60.3–64.8	1744 56.8% 54.4–59.1
IFNγ negative	bTB VL positive	Boosted IE	N RSe CI	55 83.6% 71.7–91.2	55 74.6% 61.7–84.2
IFNγ negative	bTB VL positive	Non-boosted UK, USA	N RSe CI	21 71.4% 50.1–86.2	21 66.7% 45.4–82.8
IFNγ negative	bTB VL positive	Unknown boost status IT	N RSe CI	3 66.7% –	3 66.7% –
IFNγ negative	bTB VL positive	All samples UK, IE, IT	N RSe CI	79 79.8% 69.6–87.1	79 72.2% 61.4–80.8

Diagnostic sensitivity was estimated at the high sensitivity and high specificity settings using reference sera from IFNγ positive animals showing bTB lesions at post-mortem. ^a^Boosted- samples taken within anamnestic window; non-boosted—samples taken outwith the anamnestic window (5–30 days post tuberculin injection); United Kingdom (UK), Ireland (IE), Italy (IT); United States of America (USA). ^b^N = number; RSe—relative sensitivity; CI—95% Confidence Interval.

#### IFNγ positive, visible lesion positive animals

The results show that the Rse of the test in IFNγ test positive animals with VL that were pre-boosted with PPDb was 96.9% (95% CI: 94.5–98.3%) and 95.5% (95% CI: 92.7–97.2%) respectively using the Hse and Hsp settings of the test respectively. In non-boosted samples, the figures were statistically significantly lower at 57.8% (95% CI: 50.2–65.1%) and 51.8% (95% CI: 44.3–59.3%) respectively (*P* < 0.001 in both cases). Kappa analysis using boosted samples and negative controls gave kappa values of 0.885 (95% CI: 0.860–0.910) and 0.954 (95% CI: 0.937–0.970) at the Hse and Hsp setting, indicating almost perfect agreement respectively.

#### IFNγ positive, VL negative animals

In IFNγ test positive animals without lesions that were pre-boosted with PPDb the Rse was 88.5% (95% CI: 86.3–90.4%) and 82.9% (95% CI: 80.4–85.2%) using the Hse and Hsp settings respectively. In non-boosted samples, the figures were considerably lower at 32.8% (95% CI: 29.7–36.2%) and 26.6% (95% CI: 23.9–29.8%) respectively (*P* < 0.0001 in both cases). Kappa analysis using boosted samples and negative controls gave kappa values of 0.883 (95% CI: 0.866–0.900) and 0.880 (95% CI: 0.862–0.897) at the Hse and Hsp setting, indicating almost perfect agreement respectively.

#### IFNγ negative, VL positive animals

The Rse of the test in IFNγ test negative, lesion positive animals pre-boosted with PPDb was 83.6% (95% CI: 71.7–91.2%) and 74.6% (95% CI: 61.7–84.2%) using the Hse and Hsp settings respectively. Only 21 non-boosted samples were available for testing, precluding accurate assessment of this category of sample. Kappa analysis using boosted samples and negative controls gave kappa values of 0.530 (95% CI: 0.439–0.621) and 0.742 (95% CI: 0.650–0.834) at the Hse and Hsp setting, indicating moderate and substantial agreement respectively.

### Likelihood ratio analysis

Likelihood ratio analysis was performed on Dse and Dsp data obtained using the Hse setting of the test. The results are shown in Table 7.

**Table 7:** Likelihood ratio (LR) for positive (LR +) and negative (LR−) boosted serum samples at the high sensitivity setting.

Sample categories	DSe 95% CI	Rse 96%CI	DSp 95% CI	LR + 95% CI	LR- 95% CI	DOR^a^ 95% CI
*M. bovis* culture positive/ bTB free	93.9% 89.8–97.7	–	98.4% 98.0–98.7	59.2 46.8–74.8	0.06 0.04–0.10	958 521–1761
VL positive/ bTB free	–	96.2% 95.0–97.3	98.4% 98.0–98.7	60.2 47.6–76.3	0.04 0.03–0.05	1577 1073–2317
SICCT positive/ bTB free	–	94.3% 93.1–95.3	98.4% 98.0–98.7	59.2 46.6–74.7	0.06 0.05–0.07	1011 744–1373
IFNγ positive/ bTB free	–	90.0% 88.3–91.6	98.4% 98.0–98.7	56.4 44.5–71.4	0.10 0.09–0.12	555 412–748

^a^DOR - diagnostic odds ratio.

The LR + and LR^-^ were 58.8 (95% CI: 46.4–74.6) and 0.06 (95% CI:0.04–0.10) respectively for boosted samples from *M. bovis* culture positive animals. The DOR was 953. Similar results were obtained from animals showing VL at post-mortem, where the LR + and LR^-^ were 60.2 and 0.06 respectively using boosted samples. The DOR was 1577.

In boosted samples from SICCT positive animals, the LR + and LR- were 59.2 (95% CI: 46.6–74.7) and 0.06 (95% CI: 0.05–0.07) respectively while the DOR was 1011. The LR + and LR- were 56.4 (95% CI: 44.5–71.4) and 0.10 (95% CI: 0.09–0.12) respectively for boosted samples from IFNγ positive animals while the DOR was 555. The test thus showed good ability to rule in infection (LR + results) and rule out infection (LR- results), with the high DOR value indicating good overall ability to rule in and rule out infection.

### Repeatability of the Enferplex Bovine TB antibody test

A negative sample, a weakly positive sample and a strongly positive sample were tested in quadruplicate within run, between runs, between days, between operators, and between 3 batches. The mean S/CO ratio values obtained following subtraction of the blank value for the negative sample within run, between 20 runs, and between 3 batches ranged from − 0.024 to 0.052. The CV observed for the weak positive and strong positive samples within wells ranged between 4.0 and 6.2%, and 1.3 and 3.3% respectively across the 11 antigens. Between operators, the S/CO CVs ranged from 3.4 to 6.9% and 1.3–3.8% for the weak and strong positive samples respectively. The day-to-day variation ranged between 3.6 and 6.5% and 1.3–3.8% for the weak and strong positive samples respectively. The variation between kit batches ranged between 3.5 and 6.9% and 1.1–3.5% for the weak and strong positive samples respectively. The mean values for each antigen obtained over 20 runs of the assay (over 2 days by 2 operators using 3 kit batches) did not vary more than 2 SD in 97.3% and 97.8% of results for the weak positive and strong positive samples respectively. Analysis using linear mixed-effect models showed that < 1% of the variation observed was due to the plate, kit or operator. The binary results obtained for serial batch repeatability using the two-antigen rule showed complete concordance between the Enferplex bTB test two-antigen rule result and sample category across the 3 kit batches. Overall, the within run, between run and between kit batch repeatability of the Enferplex bTB test was excellent.

### Reproducibility of the Enferplex Bovine TB antibody test

Seven negative, 7 weak positive and 7 strong positive samples were tested in duplicate using two kit batches in three independent laboratories to assess reproducibility of the Enferplex bTB test.

#### Analytical reproducibility

The mean S/CO ratio values obtained for the negative samples were all close to zero (Supplementary Fig. 1 online). Most S/CO ratio responses (63/77) obtained with weak positive samples had CVs less than 10%. There were 14 exceptions where the %CVs were > 10%. Of these, 13/14 samples were associated with responses that were below threshold for the individual antigens and would be deemed to be negative responses for those antigens. The results show that most of the S/CO ratio responses (68/77) obtained with strong positive samples had CVs less than 10%. There were 9 exceptions where the CVs were > 10%. Of these, 3/9 samples were associated with responses that were below threshold for the individual antigens and would be deemed to be negative responses for those antigens. Only 1/6 of the remaining responses had a CV > 20%. The CVs observed thus tended to reflect the various levels of S/CO ratios for individual antigens such that antigens with low S/CO ratios or S/CO ratios that were below threshold tended to give higher CVs, like those observed in the negative sample panel. Linear mixed-effect models were applied with kit batch, lab, plate and sample (as random effects) to determine how much of the variation in S/CO ratio values was due to these variables. For all antigens, < 1% of the variation was due to kit, laboratory, or plate, with 99% due to the sample. The results show a high degree of analytical reproducibility between laboratories for individual responses to the 11 antigens.

#### Diagnostic reproducibility

The results showed complete concordance between the 3 laboratories with respect to the categorisation of the positive and negative samples. The test thus shows excellent levels of analytical and diagnostic reproducibility between laboratories.

## Discussion

Work in humans with TB using whole genome arrays has indicated that the antibody response was primarily directed against around 10 recombinant antigens, all of which had been shown previously to be present in PPD^32,33^. We have used 11 antigens that are collectively recognised by a high proportion of cattle infected with *M. bovis*. The diagnostic specificity estimated using samples from bTB free animals showed a value of 98.5% and 99.7% using the Hse and Hsp settings of the test respectively. No loss in specificity was observed in bTB free animals after a single tuberculin injection. These results are consistent with other studies in the literature^36^^,^^37^^,^^38^.

Relatively small differences in specificity were observed between the samples from different countries or continents, suggesting little or no interference in test performance by or environmental mycobacteria or other infections. However, no samples were available from Asia, Africa or South America to allow diagnostic specificity to be assessed more widely.

MAP infection is common in cattle and has the potential to mask bTB infection in tuberculin skin tests due to cross-reactions between MAP and bTB antigens^9,34,35^. Such cross-reactions could potentially affect serological assays. To assess analytical specificity, samples from bTB free animals that were MAP antibody positive or MAP culture positive, or both were analysed. The results obtained were consistent with those obtained from bTB free animals without evidence of MAP infection, showing that there was no significant cross-reaction between MAP antibodies and the Enferplex bTB test antigens. A similar lack of humoral cross-reaction was observed using the Enferplex Caprine TB antibody test in goats vaccinated against MAP^31^, or when PPDa from *M. avium* is used as an antigen for serological assays in bTB infected cattle^34^.

The TT and the IFNγ test are indirect tests that detect cell mediated immune responses generated by the host T cell memory cells in *M. bovis* infected animals. In a similar manner, injection of PPDb is likely to result in re-stimulation of memory T and B cells in vivo giving rise to boosted antibody responses typical of secondary immune responses. The Dse estimates observed for the test in *M. bovis* culture positive, TT positive, or IFNγ test positive animals thus varied depending on whether the samples were taken during the anamnestic window following PPDb boosting or not. The results described here are consistent with those described in the literature in relation to anamnestic serological responses following PPDb injection of infected animals^19,21,24–27^.

The Dse estimates in *M. bovis* culture positive boosted animals was 93.5% at both the Hse and Hsp setting. The test thus detects most truly infected animals. This is consistent with the view that injection of PPDb antigen stimulates anamnestic antibody responses and induces the Koch phenomenon characterised by a DTH reaction causing dissolution of granulomas and release of bacterial antigens^50^ resulting in additional antibody production. The reason for the test not detecting the remaining 6.5% of *M. bovis* culture positive animals is not known. The Koch phenomenon has not been closely studied in cattle, and its extent and timescale in this species is not known. The observation that these animals showed no evidence of antibody production even after boosting suggests a lack of *M. bovis* primed B cell memory cells in blood despite the animals being SICCT and *M. bovis* culture positive. It is recognised in human TB that granulomas which are well established, particularly if circumscribed with a fibrous capsule, appear to be difficult to dissolve despite a vigorous DTH reaction^50^. The bacteria in such granulomas tend to be quiescent or dormant resulting in low antigen release. In cattle, acid-fast bacteria are difficult to detect in some animals despite the presence of florid lesions, suggesting that the bacterial load is low and consequently the level of bacterial antigen would also be low. Such bacteria often require application of resuscitation techniques before the they will grow in vitro*.* This, potentially, could result in some animals being *M. bovis* culture positive without showing evidence of antibody production.

Similarly, high Rse estimates using the two sensitivity settings of the test (93.6% and 91.1% respectively) were observed in SICCT positive boosted animals, though some SICCT positive animals were not detected. The latter may have been due to T cell control of *M. bovis* replication curtailing antigen secretion that would be required for antibody responses to be generated.

The Rse estimates using the IFNγ test as the comparator were 90.3% and 85.9% in boosted animals using the Hse and Hsp settings of the test respectively. The lower estimates obtained compared to the SICCT may reflect the lower specificity of the IFNγ test^9,12,15,16^, such that some of the IFNγ positive samples may have been false positives. Also, some animals may have been in the early stages of infection when IFNγ responses could be dominant over antibody responses^39,40^.

Animals showing inconclusive TT reactions are problematical for accurate bTB diagnosis. In the UK, animals showing two consecutive inconclusive reactions in short interval testing are regarded as being infected and are slaughtered. When tested in the Enferplex bTB test, a high proportion of both 2 × IR and 1 × IR animals gave positive reactions, giving confidence that these animals were infected with *M. bovis*. The test was positive in all VL/*M. bovis* culture positive boosted 2 × IR and 1 × IR animals. The results indicate that the Enferplex bTB test detects SICCT inconclusive infected animals which in the case of 1 × IR animals are currently not being removed to slaughter. The results suggest that the test could be useful in resolving the infection status of IR animals, thereby removing SICCT or IFNγ negative infected animals earlier and improving the efficiency of bTB control and eradication.

The diagnostic accuracy of the test was also assessed in relation to the presence or absence of lesions following post-mortem using the Hse and Hsp test settings. High Rse and Dsp estimates of 97.1% and 96.3% respectively were observed using samples from TT positive, bTB lesion positive boosted animals. Strong correlations between antibody levels and the presence of lesions in post-mortem tissues have been described in the literature^9,39,40^ and the results are thus consistent with these reports.

Antibody positive animals were also found in TT positive animals with no VL, an observation not unexpected since lesions can be missed at post-mortem examination with typically only a single VL being found in herds subject to eradication schemes^41^. For example, the vast majority (84.7%) of the UK animals with VL had only a single VL [APHA bTB database]. It is even more difficult to detect histological lesions in VL negative animals due to the lack of information of where to sample in the tissues. Nevertheless, histological lesions, positive *M. bovis* cultures, and/or positive PCR reactions have been described in 10–47% of animals with no VL^42–44^. The positive antibody results found in this study provide confirmatory evidence that the TT positive animals without lesions were infected with *M. bovis*. Also, animals without lesions have been shown to shed *M. bovis* following experimental infection^45,46^ suggesting that they are epidemiologically important and a significant risk to the rest of the herd.

In addition, the antibody test detected 88.2% of TT negative, lesion positive boosted animals. Similar results were obtained in IFNγ test negative, lesion positive animals, where 83.6% of boosted animals were antibody positive. These results are likely to reflect the variable sensitivity of the SICCT (51–85% for standard interpretation) and IFNγ test (67.0–85.8%) reported in the literature^5^. In chronically infected herds, latent class analysis gave sensitivities for standard interpretation of SICCT between 40.5 and 57.7%^4^. This low sensitivity may reflect the development of a state of anergy in advanced disease^47^. The results thus show that the Enferplex bTB test can detect infected animals missed by the TT or IFNγ test. This is not surprising since reciprocal cell mediated and humoral responses have been documented^38,39^, and may reflect swings between type 1 and type 2 T helper cell responses during infection^39^. However, relatively few samples of SICCT negative, VL positive and IFNγ test negative, VL positive animals were available and larger sample sets are required to give a more accurate estimate of diagnostic sensitivity in these categories of infected animals. A further limitation was the lack of samples from outside the EU and the USA which will be required to determine the results found here can be generalised. The Enferplex bTB test results obtained from *M. bovis* culture positive, SICCT positive or IFNγ positive animals underwent likelihood ratio analysis to assess the strength of the evidence they provided for *M. bovis* infection being either present or absent^48,49^. The Enferplex bTB test showed LR results provide good diagnostic evidence of the infection being either present (LR + 58.8 for culture positives: LR + 60.2 for VL) or absent (LR − 0.06 in both cases for samples from bTB negative animals), DOR was 953 and 1577 respectively. These results compare with LR^+^  = 32.3 (95% CI: 25.4–40.9); LR^-^ = 0.361 (5% CI: 0.314 0.412); DOR 89.4 for the IDEXX test, and LR^+^  = 32.5 (95% CI: 6.6–125.7); LR^−^ = 0.158 (95% CI: 0.088–0.285); and DOR 205.8 for Bovigam test^49^.

Overall, the results indicate that the Enferplex bTB test at the Hse or Hsp setting could be used to detect antibodies to *M. bovis* in serum from bTB-infected cattle to aid eradication and/or in surveillance schemes to detect primary infection in conjunction with other methods. For example, the test could be used to confirm a diagnosis of bTB in suspect cases, detect bTB infected animals missed by SICCT or IFNγ tests, or to aid resolving infection status of animals showing an inconclusive reaction in a TT test. Other potential uses of the test include pre- and post-movement testing, or in serological surveys to estimate prevalence of infection. In addition, the test could provide a valuable tool for slaughterhouse surveillance.

However, it should be noted that the test has only been assessed in samples from European countries and the USA and that further studies are required to determine Dse and Dsp in other continents with different prevalence and *M. bovis* clades.

## Methods

### Study design

The study aimed to determine diagnostic sensitivity and specificity by testing defined positive and negative reference sera from animals known to be infected with *M. bovis* or known to be from herds free of bTB respectively. All methods were performed in accordance with the relevant national and international guidelines and regulations and complied with ARRIVE guidelines. Reference sera from animals naturally infected with *M. bovis* and bTB free animals over 6 months of age from the EU and USA were tested. The positive reference sera were obtained from bTB infected animals defined by culture of *M. bovis* from post-mortem tissues (Definitive Positive Reference Standard), and by results obtained in tuberculin tests (SICCT/CFT) and the IFNγ test (Comparative Positive Reference Standards). The negative reference sera were obtained from herds that were negative in SICCT/CFT, and/or were from countries or regions that held Officially Tuberculosis Free (OTF) status and had no recent history of bTB or contact with animals likely to have been exposed to *M. bovis*. Relevant subsets of animals with bTB were also analysed. These included animals positive by TT or IFNγ tests with or without visible lesions at post-mortem. Samples taken 5–30 days post TT were regarded as being ‘boosted’ for antibody, while those taken outside this anamnestic window were regarded as being ‘non-boosted’. The samples obtained encompassed wide variations in geographical location, cattle breed, age, husbandry, farm practices, and farm management. Antibodies to *Mycobacterium avium* subsp. *paratuberculosis* (MAP) were measured using the ID.vet ID Screen Paratuberculosis Indirect ELISA or IDEXX MAP antibody test.

## Reference samples

### Negative reference serum samples

Serum samples from negative reference animals (n = 4747) were obtained from the United Kingdom (UK), Ireland (IE), Netherlands (NL), Switzerland and Liechtenstein (CH/LI), Norway (NO), Spain (ES), France (FR) and the USA, summarised in Table 1. Samples were obtained retrospectively as remnants from serum banks held by regulatory authorities or various institutions following routine diagnostic testing from animals tested for non-tuberculosis diseases (MAP, BVDV, IBR, FH, BCV, BRSV) in several counties (UK, IE, NL, NO, ES, CH/LI). These samples and data were collected by local veterinary surgeons for routine diagnostic purposes which is regulated by the national and EU legislation.

Most of samples were from females (3012/3183 gender records, 94.6%). There were 40 breeds/crosses included in the study. The samples were from herds in low-risk areas or countries with OTF status that had been free of bTB for several years as based on routine surveillance screening by SICCT, CFT, IFNγ test or post-mortem examination. In the case of samples from the UK, herds had been free of bTB for at least 8 years, and no herds within 10 km had recorded bTB in the previous 12 months. A further set of 339 sera from 5 herds sampled 5–30 days after the SICCT from bTB free herds in the UK and FR were available as anamnestic specificity controls. Samples from IE were obtained from herds in the low cattle bTB prevalence areas defined as those herds with 2 or fewer standard SICCT reactors in the annual round of skin testing over the previous preceding 5 years (Greenfield sites)^51^.

Animals from low-risk areas or from OTF countries that were MAP culture and/or MAP antibody positive were included in the negative reference set. The blood samples were taken at the time of reading the TT, and therefore were “non-boosted”. The details are shown in Table 1.

### Positive reference serum samples

Most positive references samples from bTB infected animals were obtained retrospectively as remnants from serum banks held by regulatory authorities (UK Animal and Plant Health Agency, the United States Department of Agriculture, the Irish Department of Agriculture and Marine, and the IZLER Biobank, Instituto Zooprofilattico Sperimentale della Lombardia e dell’Emilia, IT). Some positive reference samples were obtained following regulatory approval at the abattoir post slaughter.

The majority of samples were from females (3333/3979 gender records; 83.7%). There were 40 breeds/crosses included in the study. The animals were positive by one or more of the following criteria: SICCT, SICT, CFT, IFNγ test, VL, or *M. bovis* culture. Sera from animals showing two consecutive inconclusive reactions in the SICCT (2 × IR) were included as positive reference samples (APHA Official Veterinarian instructions available from: http://apha.defra.gov.uk/External_OV_Instructions/TB_Instructions/Skin_Test/Skin_Test_Day_Two.html). Animals showing a single IR reaction were also included for comparative purposes. Typically, some of these animals go on to show an IR reaction at the next short interval test and become a 2 × IR category animal. Of 131 UK SICTT positive animals with VL, 111 (84.7%) registered a single VL, 17 (13.0%) had 2 VLs and 3 (2.6%) had 3 VLs (APHA bTB database).

Samples were taken approximately 5–30 days post PPD injection when anamnestic antibody responses were likely to be developing or optimal (termed ‘boosted’) or outside this time frame when anamnestic responses were unlikely to be present (termed ‘non-boosted’). The details are summarised in Table 2.

In this study, a total of 4,747 negative reference samples were used to determine test specificity, while a total of 4,718 positive reference samples were used to determine test sensitivity: 482 *M. bovis* culture animals, 2651 TT positive animals, and 2753 IFNγ positive animals were used to determine test sensitivity.

### Enferplex Bovine TB antibody test description

The antigens used in the study were as follows: Rv2975 synthetic peptide p6^52^; PPDb; recombinant Rv2873; recombinant Rv2975; Bovine TB cocktail; recombinant Rv2031c-Rv1886c fusion protein; recombinant Rv3875-Rv3874 fusion protein; recombinant Rv3874-Rv3875 fusion protein; recombinant Rv2626; recombinant Rv0251c; recombinant Rv2031c.

Rv2975 peptide p6 and PPDb were obtained from Genosphere Biotechnologies, Paris, France and Prionics Ag, Switzerland respectively, while the other antigens were obtained from Lionex (Braunschweig, Germany) and Genscript Biotech (Netherlands). These antigens were chosen on the basis that they give high operational specificity using the two-antigen rule while individually providing incremental additions to the overall Dse sensitivity of the test.

The antigens were deposited in a multiplex planar array as individual 30 nl spots into wells of 96 well black polystyrene microtiter plates using BioDot aspirate/dispense platforms. Plates were blocked, stabilised, dried and stored at 2–8 °C until use. The assay was carried out as previously described^28,31^, with the following modifications. Serum samples were diluted 1:200 into sample dilution buffer (Enfer Buffer B, Enfer Scientific) and mixed before 50ul was added per well. The plates were incubated at 37 °C for 60 min with agitation (900 rpm). The plates were washed 6 times with 1X Wash (Enfer Wash buffer, Enfer Scientific) and aspirated. The detection antibody, sheep anti-bovine IgG—HRP (Bethyl Laboratories) was prepared to 1:20,000 dilution. The plates were incubated at 37 °C for 60 min with agitation (900 rpm). The plates were washed as above and 50 ul of prepared chemiluminescent substrate (50:50 dilution of substrate and diluent), (Advasta, USA) was added per well. Relative light units (RLU) were captured (220 s exposure) immediately, using Quansys Biosciences Q-View™ LS imager and Q-View™ software (v 3.1). The results were defined using the Enferplex Bovine TB Macro, based on individual antigen thresholds after subtracting the RLU value obtained from a blank spot. The individual antigen thresholds were set using known positive and negative serum samples. Individual antibody levels were determined by calculating the signal/cut-off (S/CO) ratio for each antigen. The performance levels indicated in this study were based on multiple batches of the Enferplex bTB test and reflect the biological diversity with respect to kit components (antigens, buffers, conjugates, positive and negative controls). The thresholds for individual antigens in the Enferplex bTB test were selected empirically. Two threshold settings were established by selecting individual antigen cut-offs appropriately to give a Hse setting with a target specificity of 98.0%, which optimises the test for eradication purposes and a Hsp setting with a target specificity of 99.5%, which optimises the test for screening purposes. Thus, the higher the sensitivity the lower the specificity and vice versa.

The threshold for overall assay positivity was set based on a two—antigen rule, whereby the RLU signals from two or more antigens need to be above their individual antigen thresholds for the sample to be registered as “positive”^28^. Thresholds for individual antigens were set between 95.1 and 99.8% specificity based on true negative reference sera to give the “Hse” setting using the two-antigen rule. Thresholds for individual antigens were set between 97.3 and 99.9% specificity based on true negative reference sera to give the “Hsp” setting for the test using the two-antigen rule. An ‘Enferplex Bovine TB Macro’, automatically calculates the results for Hse and Hsp.

### Repeatability and reproducibility trials

To determine the within-run, between-run, and between-batch variation, three categories of sera were used to test within run repeatability: one serum sample that was negative against all 11 antigens; one serum dilution for each antigen giving strong positivity; one serum dilution for each antigen giving weak positivity. The samples were run in quadruplicate over 20 runs, split between two days and two operators for three kit batches. For reproducibility studies, an evaluation panel of serum samples comprising negative, weak positive and strong positive serum samples were blinded and sent to two independent laboratories for analytical reproducibility testing. Seven negative samples, seven weak positive samples, and seven strong positive samples (based on the two-antigen rule) were tested blind in duplicate using two plates from two different kit batches and one technician in the Enfer laboratory (Enfer Scientific laboratory, IE) and each of the two independent laboratories: Animal and Plant Health Agency (APHA), Weybridge, UK; Molde Mastitis Reference Laboratory, TINE, NO. The results from the Molde and Weybridge laboratories were sent to Enfer Scientific for un-blinding and analysis.

Data were expressed as mean, SD, and 95% Confidence interval. Differences in proportions were assessed using Fisher’s Exact test. The degree of agreement between Enferplex test and positive and negative reference comparators was assessed using Cohen’s Kappa analysis. Likelihood ratios were calculated using MedCalc statistical programme. For the repeatability and reproducibility analyses, a series of linear mixed-effect models were run with kit batch, laboratory, microtitre plate and sample were entered as random effects. These models also permitted the amount of variation explained by batch, laboratory, microtitre plate and sample to be assessed via calculation of intra-class correlation coefficients (ICCs). The results included the overall means, SD, coefficient of variation (CV) defined as the ratio of the SD to the mean expressed as a percentage, and 95% CI and an estimate of how much variation was due to these variables. The analyses were carried out in R (v3.51, (C) 2018 The R Foundation for Statistical Computing), using the lme4 (v1.1–18.1), lmerTest (v3.0–1) and sjPlot (v2.6.0) and sjstats (v0.17.0) packages.

## Supplementary Information

Supplementary Information 1.

Supplementary Information 2.

Supplementary Information 3.

## Data Availability

All data generated or analysed during this study are included in this published article and its supplementary files.
