# Supplementary material for: Diagnostic accuracy of the Enferplex Bovine Tuberculosis antibody test in cattle sera
Source: Sci Rep. 2023 Feb 1;13:1875. doi: 10.1038/s41598-023-28410-9 (PMC9892036; doi:10.1038/s41598-023-28410-9)

Diagnostic accuracy of the Enferplex Bovine Tuberculosis antibody test in cattle

Amanda O'Brien, John Clarke, Alastair Hayton, Andy Adler, Keith Cutler, Darren J. Shaw, Clare Whelan, Neil J. Watt, Gordon D. Harkiss

Supplementary Fig. S1

Reproducibility of the Enferplex Bovine TB antibody test

An evaluation panel of serum samples comprising negative, weak positive and strong positive serum samples were blinded and sent to two independent laboratories for analytical reproducibility testing. Seven negative samples, seven weak positive samples, and seven strong positive samples (based on the two-antigen rule) were tested in duplicate using two plates from two different kit batches (Kit A and Kit B) and one technician in the Enfer laboratory (Enfer Scientific laboratory, IE) and each of the two independent laboratories: Animal and Plant Health Agency (APHA), Weybridge, UK; Molde Mastitis Reference Laboratory, TINE, NO. The results from the Molde and Weybridge laboratories were sent to Enfer Scientific for un-blinding and analysis.

The results for each antigen in each category of sample expressed either in raw unblanked relative light units (RLU) or in blanked RLU/threshold (Signal/cut-off) ratios for Kit A and Kit B and Plate 1 and Plate 2 are shown. Enfer – ●; Norway – ●; Weybridge – ●.

a) Negative samples − raw

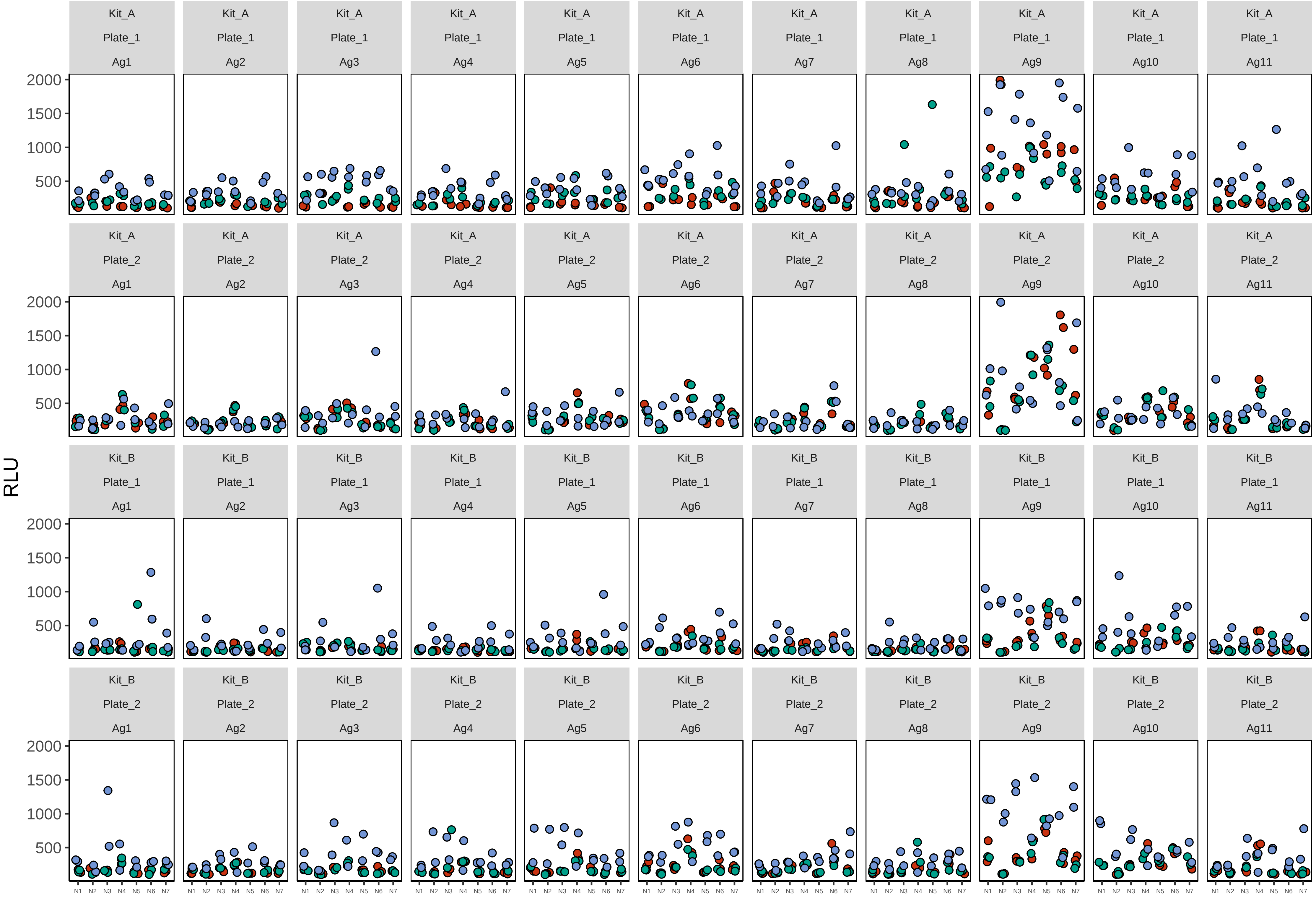

b) Weak positive samples − raw

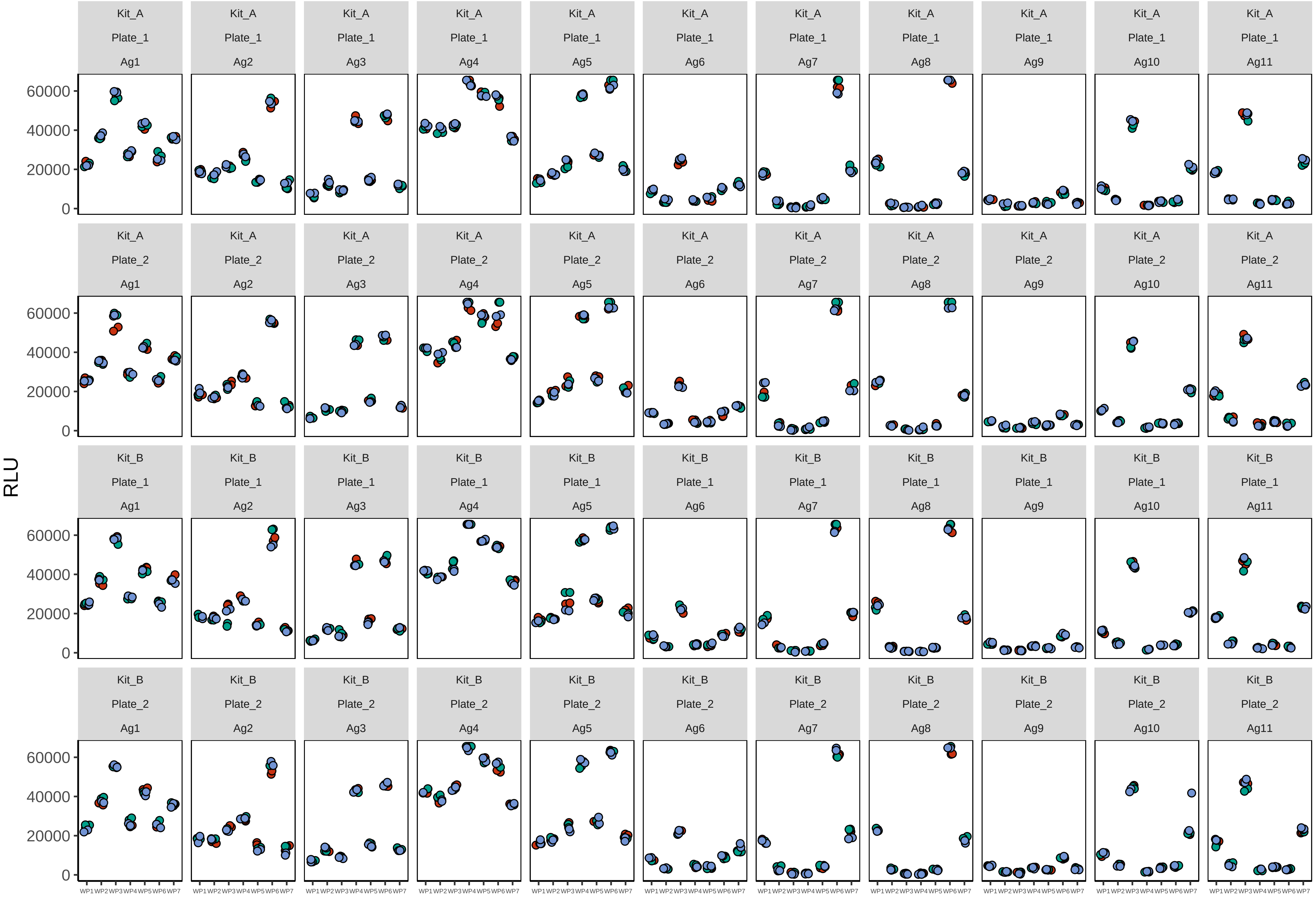

c) Strong positive samples − raw

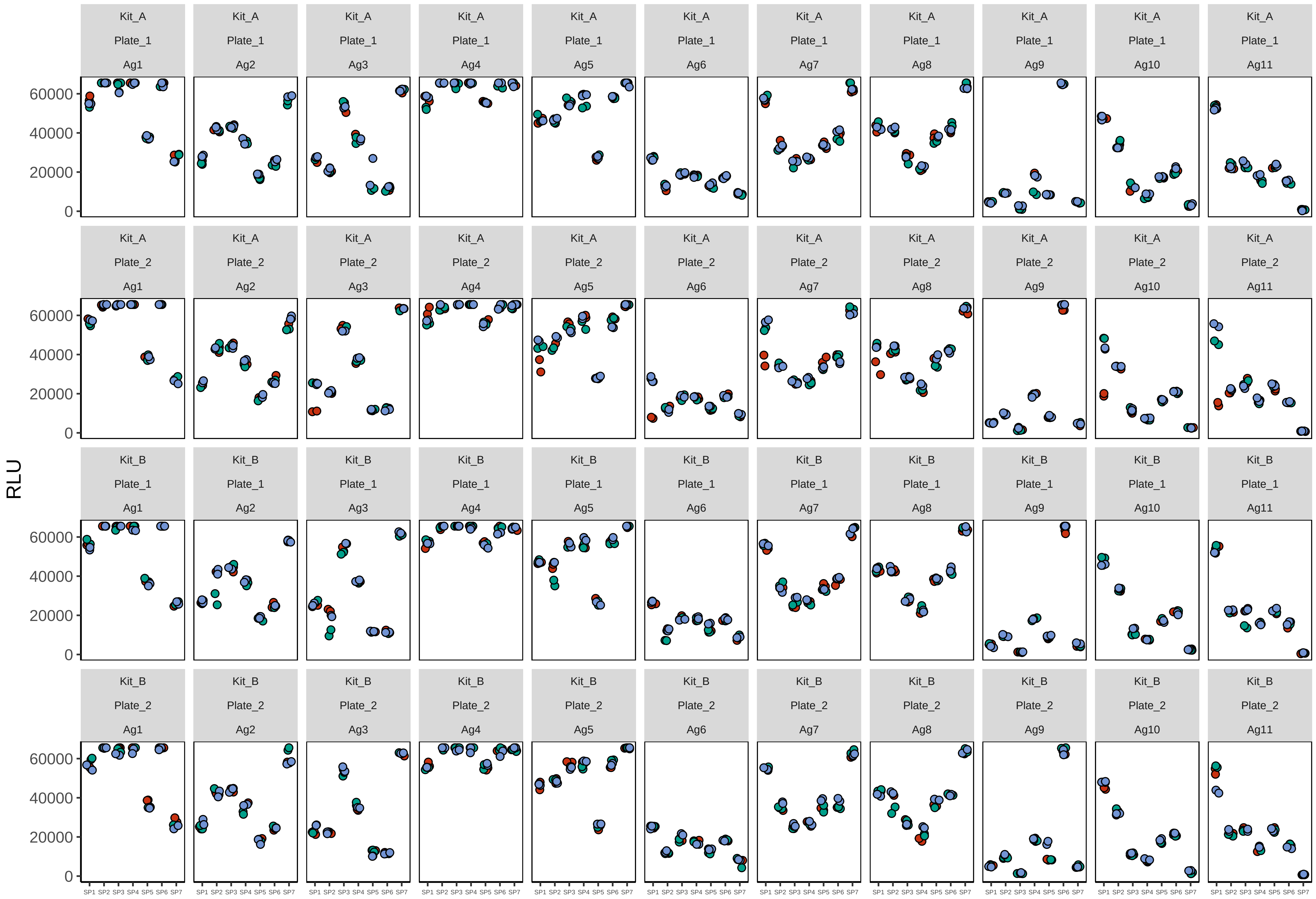

d) Negative samples − signal

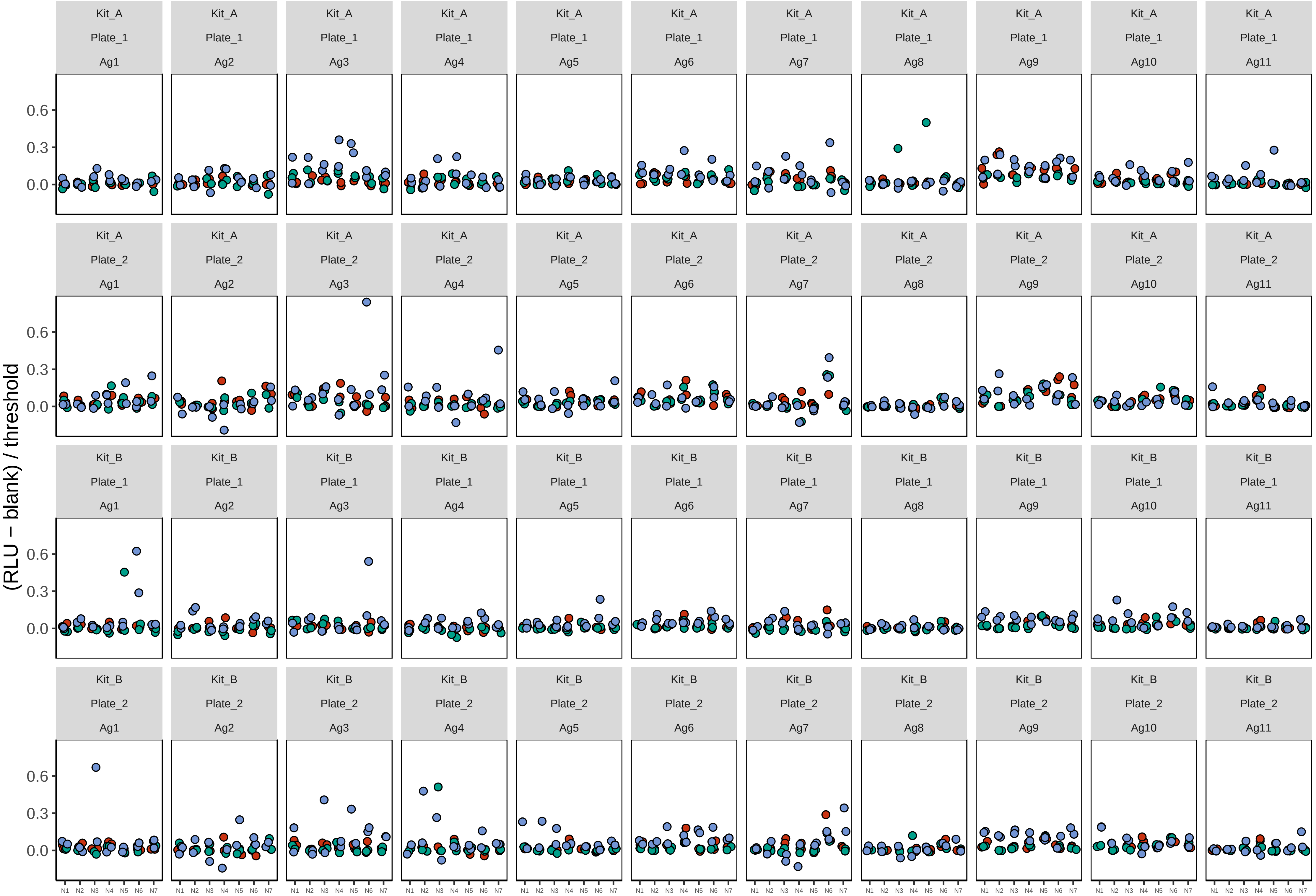

e) Weak positive samples − signal

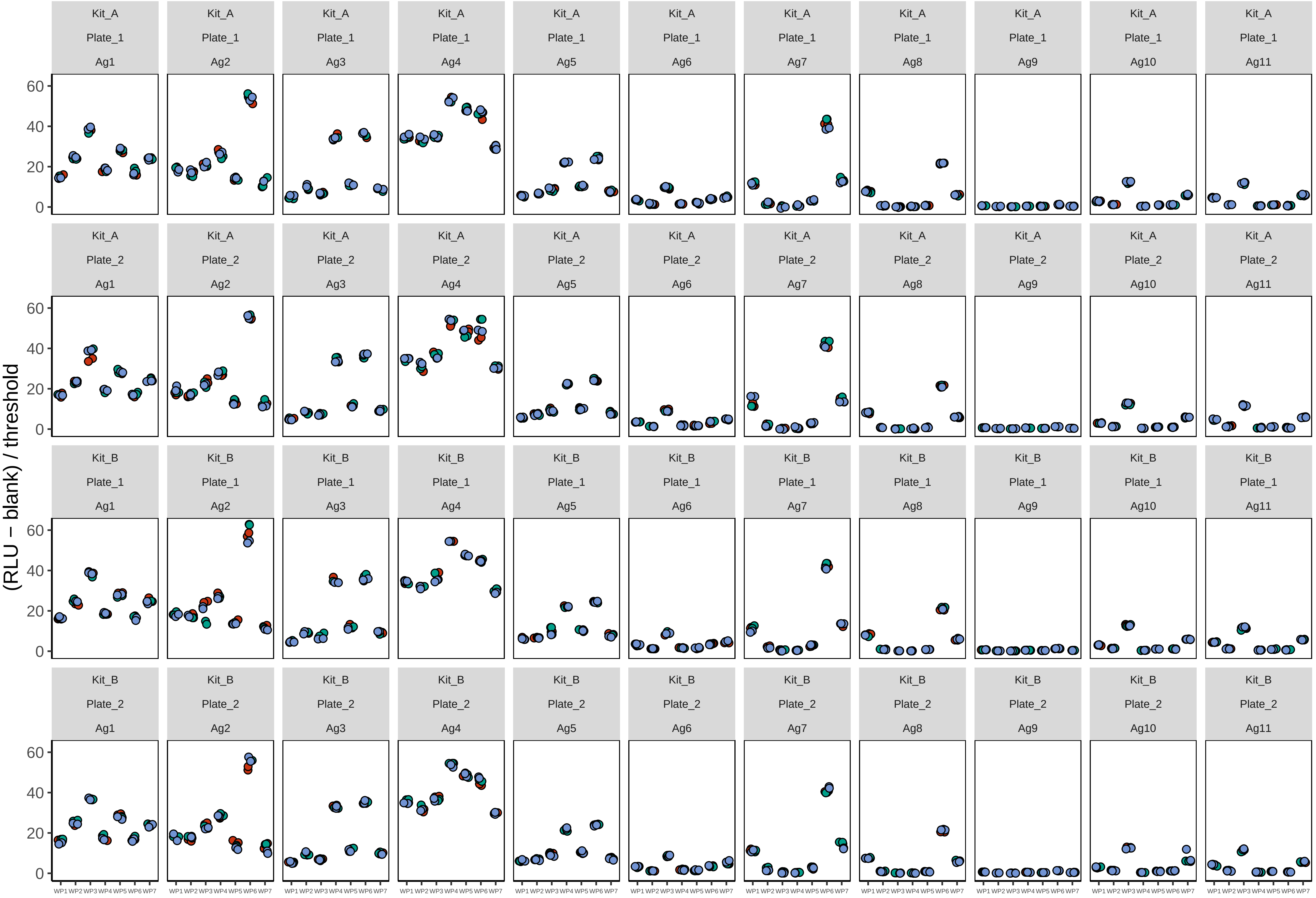

f) Strong positive samples − signal

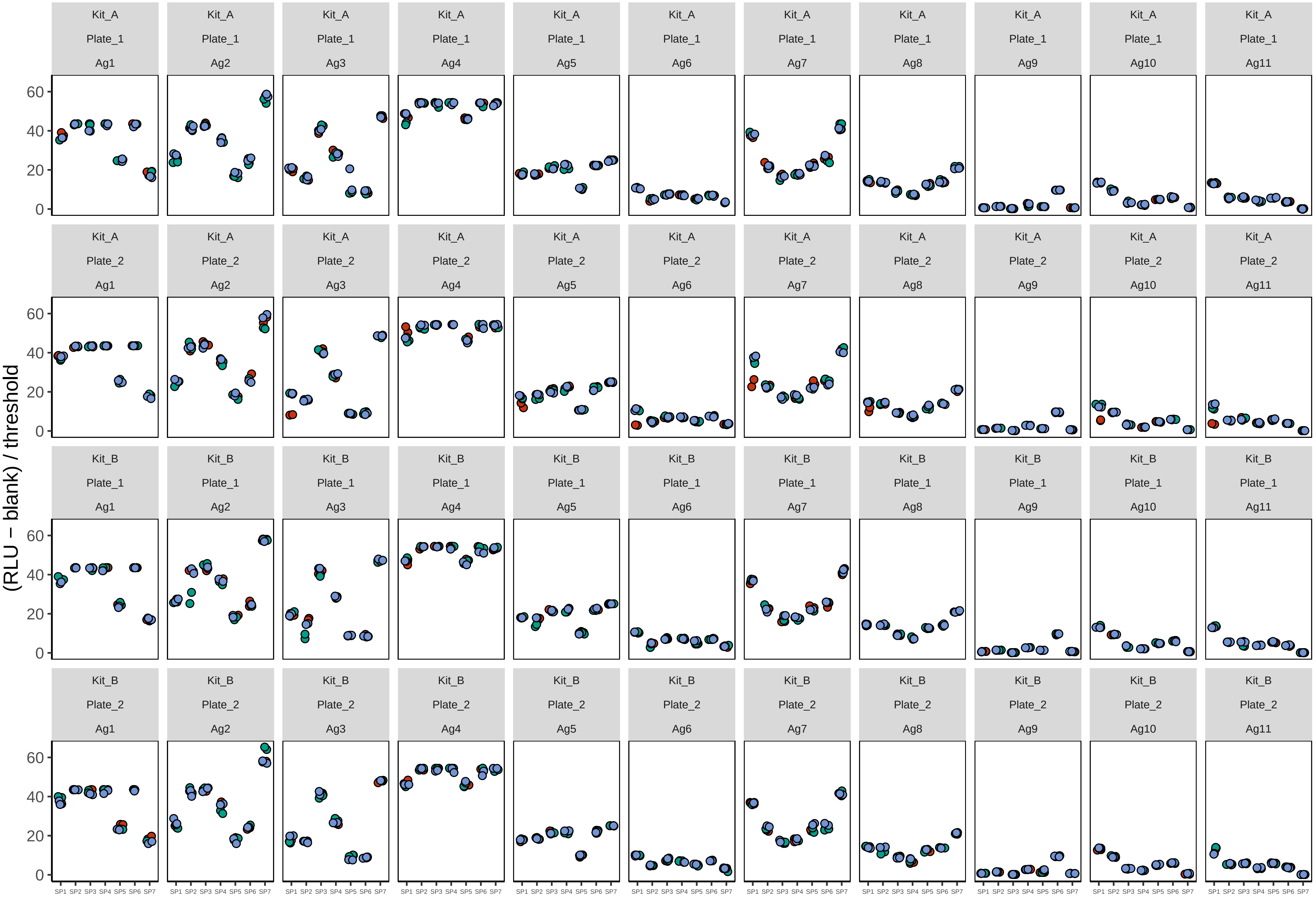

Supplement: Supplementary file 1 — Supplementary Information 1. [file 41598_2023_28410_MOESM1_ESM.pdf]
