# Supplementary material for: Diagnostic accuracy of the Enferplex Bovine Tuberculosis antibody test in cattle sera
Source: Sci Rep. 2023 Feb 1;13:1875. doi: 10.1038/s41598-023-28410-9 (PMC9892036; doi:10.1038/s41598-023-28410-9)
Supplement: Supplementary file 3 — Supplementary Information 3. [file 41598_2023_28410_MOESM3_ESM.docx]

**Diagnostic accuracy of the Enferplex Bovine Tuberculosis antibody test in cattle**

**Amanda O’Brien^1^, John Clarke^1^, Alastair Hayton^2^, Andy Adler^2^, Keith Cutler^2^, Darren J. Shaw^3^, Clare Whelan^1^, Neil J. Watt^4^, Gordon D. Harkiss^4^**

^1^Enfer Scientific, Unit T, M7 Business Park, Newhall, Naas, County Kildare, Ireland.

^2^SureFarm Ltd, The Transmission Hall, Rampisham Business Centre, Rampisham Down, Maiden Newton, Dorset, DT2 0HS, UK.

^3^Royal (Dick) School of Veterinary Studies & The Roslin Institute, University of Edinburgh, Easter Bush Campus, Edinburgh UK.

^4^MV Diagnostics Ltd, Roslin Innovation Centre, University of Edinburgh, Easter Bush Campus, Edinburgh, UK.

**Supplementary Table S2**

**Diagnostic specificity of the Enferplex Bovine TB antibody test using serum samples from different countries.**

| **Sera from bTB free animals** | | | **Specificity** | | | |
| --- | --- | --- | --- | --- | --- | --- |
|  |  |  | **High sensitivity setting** | | **High specificity setting** | |
| **Sample source** | **Category of animal** | **Number of animals** | **%** | **95% CI** | **%** | **95% CI** |
| **UK** | SICCT negative OTF herds | 2279 | 98.1 | 97.4-98.6 | 99.8 | 99.5-99.9 |
| **IE** | SICCT negative, IFNγ negative herds | 643 | 98.1 | 96.8-98.9 | 99.8 | 99.1-100.0 |
| **CH/LI** | SICCT negative OTF herds | 554 | 98.7 | 97.4-99.4 | 98.9 | 97.7-99.5 |
| **NO** | OTF herds, OTF country, no recent history of bTB | 438 | 99.5 | 98.8-100 | 100 | - |
| **NL** | OTF herds, OTF country, no recent history of bTB | 100 | 98.0 | 95.3-100 | 100 | - |
| **ES** | OTF herds, OTF country, no recent history of bTB | 10 | 100 | - | 100 | - |
| **USA** | CFT negative OTF herds | 381 | 99.2 | 97.7-99.7 | 99.5 | 98.1-99.9 |

Diagnostic specificity was estimated at the high sensitivity and high specificity settings of the test using reference sera from bTB free animals in UK, IE, CH/LI, NO, NL, ES, and USA. SICCT – single intradermal comparative cervical tuberculin test; CFT – caudal fold test; OTF – officially tuberculosis free. CI – confidence interval.
